# Serum Carbohydrate Antigen 19-9 in Differential Diagnosis of Benign and Malignant Pancreatic Cystic Neoplasms: A Meta-Analysis

**DOI:** 10.1371/journal.pone.0166406

**Published:** 2016-11-11

**Authors:** Shaobo Cao, Ya Hu, Xiang Gao, Quan Liao, Yupei Zhao

**Affiliations:** Department of General Surgery, Peking Union Medical College Hospital and Chinese Academy of Medical Science, Beijing, 100730, China; University of California San Francisco, UNITED STATES

## Abstract

**Background:**

Using serum carbohydrate antigen 19–9 (CA 19–9) in discriminating between benign and malignant pancreatic disease remains controversial. We aim to evaluate the diagnostic value of serum CA 19–9 in predicting malignant pancreatic cystic lesions.

**Methods:**

Eligible studies were identified through searching MEDLINE and EMBASE prior to March 2016. Studies were assessed for quality using the Quality Assessment for Studies of Diagnostic Accuracy, 2nd version (QUADAS-2). Pooled sensitivity and specificity with 95% confidence interval (CI) were calculated using random-effects models. Summary receiver operator characteristic (SROC) curves and the area under curve (AUC) were performed.

**Results:**

A total of thirteen studies including 1437 patients were enrolled in this meta-analysis. The pooled sensitivity and specificity were 0.47(95% CI: 0.35–0.59), and 0.88(95% CI: 0.86–0.91), respectively, and the AUC was 0.87(95% CI, 0.84–0.90). Meta-regression analysis showed that sample size, region and reference standards were not the main sources of heterogeneity.

**Conclusions:**

Serum CA 19–9 has satisfying pooled specificity while poor pooled sensitivity for discriminating benign from malignant PCNs. It deserves to be widely used as complementary to other clinical diagnostic methods.

## Introduction

Pancreatic cystic neoplasms (PCNs) include serous cystic adenomas (SCAs), cystic neuroendocrine tumors, mucinous cystic neoplasms (MCNs) and intraductal papillary mucinous neoplasms (IPMNs). SCAs is a benign type of PCNs that rarely get malignant but MCNs and IPMNs may be premalignant or malignant.[1,2] The differentiation of benign and malignant PCNs can be complex preoperatively, despite the advances in imaging technology. Close follow-up was generally sufficient for some patients with benign PCNs considering the high rate of complications associated with surgical resection and the increased hospital costs.[3] Careful non-operative management seemed to be safe and effective in some asymptomatic patients.[4] Nevertheless, neoplasms with high malignant potential were supposed to be referred for surgical invention and these group of patients may be associated with poor survival even after radical resection in some cases.[5–7]Therefore, accurate and rapid diagnosis of malignant PCNs is crucial for the optimal management.

Traditional cross-sectional imaging examinations have a limited ability to differentiate benign from premalignant or malignant PCNs.[8,9] EUS-guided fine needle aspiration (FNA) is invasive and positron emission tomography (PET) is costly for many people. Laboratory tests such as the assessment of tumor markers are considered as the most frequent, economic, convenient and rapid method. The role of serum CA 19–9 in distinguishing between benign and malignant disease remains controversial. So we conducted this meta-analysis to evaluate the diagnostic precision of serum CA 19–9 in discriminating benign from malignant PCNs.

## Methods

### Article search strategy

A literature search for relevant articles including MEDLINE and EMBASE was carried out to identify eligible English-language studies published prior to March 2016 by two independent reviewers independently. The following terms were used as keywords: “CA19-9 Antigen”, “pancreas” OR “pancreatic cystic lesion” OR “Pancreatic cystic neoplasms”, “diagnosis”. The references of identified articles and review articles were also reviewed to involve more relevant studies.

### Study eligibility and quality assessment

Two reviewers (Shaobo Cao and Ya Hu) independently reviewed the searches and evaluated every article. Titles and abstracts were considered to identify the potentially eligible study. And then full text was obtained for further evaluation. Studies were included if (1) they attempted to determine the benignity or malignancy of PCNs; (2) sufficient information were provided to complete the 2×2 contingency tables; (3) histopathology results and/or clinical follow-up were used as the reference standard; (4)they were published as full-text articles. The exclusion criteria were as follows: (1) studies with insufficient data to construct 2×2 contingency tables; (2) editorial, case reports, letter to editors, comment, brief communication or meeting abstract without publication of full article; (3) included patients less than ten; (4) studies in which the relevant data overlapped with that of other studies due to patient overlap. QUADAS-2 tool was used to assess the quality, applicability, and risk of bias of included studies.[10] All the authors discussed their evaluation and any disagreement was resolved through discussion.

### Statistic analysis

Rev Man 5.3 (The Nordic Cochrane Centre, The Cochrane Collaboration, 2014) and STATA version 12.0 (STATA Corporation, College Station, Texas, USA) were used to meta-analyze the data. Pooled sensitivity, specificity, summary receiver operating characteristic (SROC) curve, diagnostic odds ratio (DOR) and the area under the curve (AUC) were calculated following a bivariate mixed-effects regression model with MIDAS tool.[11] An AUC close to 1 reflected a well-performing diagnostic precision, and a poor performance has an AUC close to 0.5.[12] Forrest plots of each study and pooled estimates for sensitivity and specificity with 95% confidence intervals (95% CI) were presented.

The Cochran's Q and I^2^ statistics were used to assess the extent of heterogeneity among the included studies.[13] The studies were considered to be with statistically significant heterogeneity if *P*<0.1 and I^2^>50%. Deek funnel plot asymmetry test was used to evaluate the potential publication bias, and *P*<0.1 was considered to be of a significant publication bias statistically. Subgroup analysis and meta-regression were applied to identify the sources of heterogeneity across studies.[14] *P* < 0.05 was determined to be statistically significant.

## Results

### Search and selection of the studies

**Fig 1:**
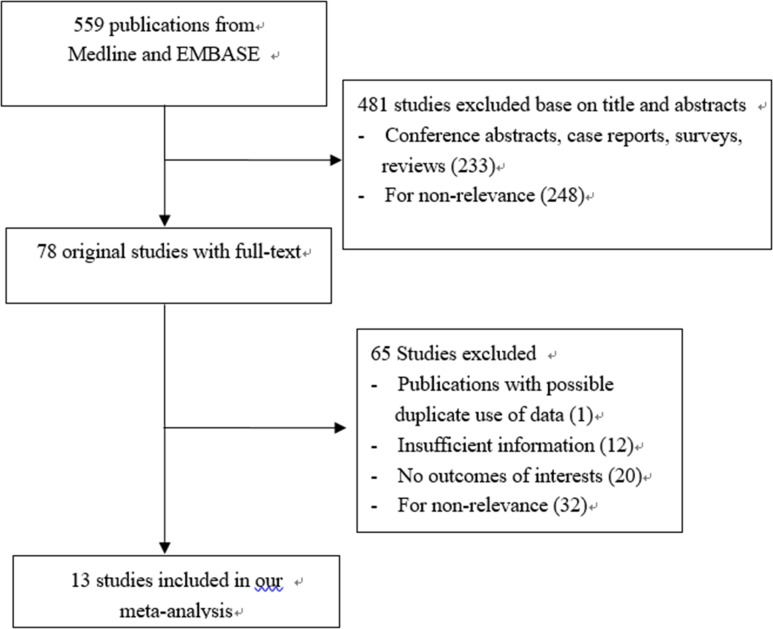
The flow chart of systematic studies search and selection procedure.

The procedure of studies search and selection was outlined in Fig 1. The systematic database search returned 559 articles. Of these, 481 articles were excluded based on the titles and abstracts. Full manuscripts from the remaining 78 articles were viewed. Finally, a total of 13 studies were eligible for data extraction and analysis.[15–27]

### Patient characteristics and quality of studies

**Fig 2:**
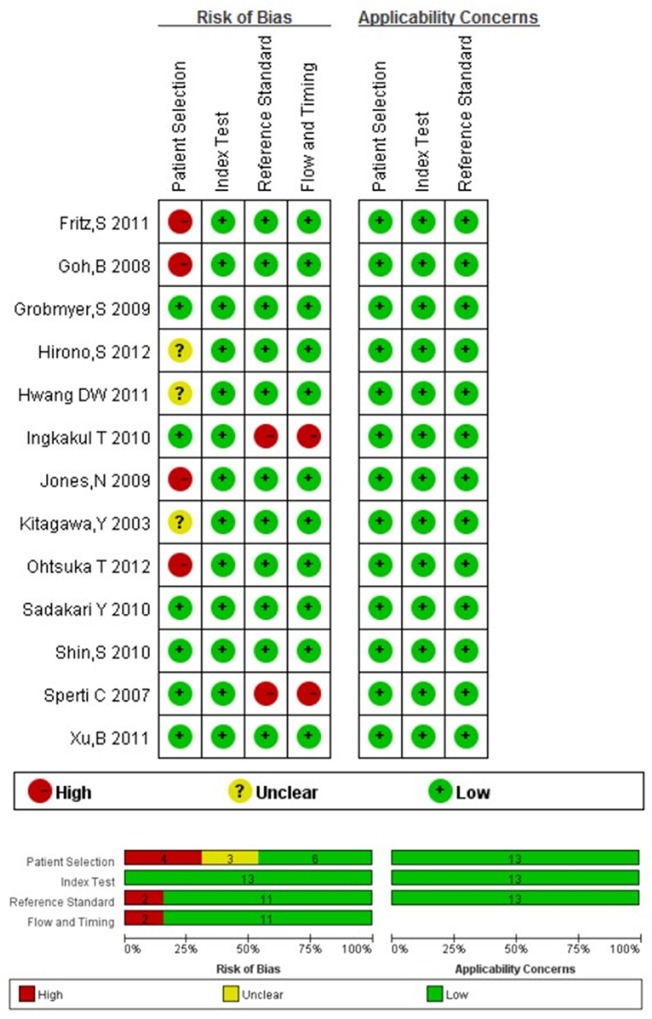
Risk of bias of each included studies with QUADAS-2 quality assessment tools.

A total of 13 studies (1 prospective, 12 retrospective) comprising 1437 patients were incorporated in this analysis, which included 948 benign lesions and 489 malignant lesions. The principal characteristics of these included studies were outlined in Table 1. The cutoff value of serum CA 19–9 used to differentiate benign and malignant cysts ranged from 35 to 45 U/ml in 11 papers, while definite cutoff value was not reported in the rest two studies.[18,19] Most of the patients underwent operations and the pathological results were available, but a part of patients were observed without surgery in two studies.[23,25] Eleven studies reported that elevated serum CA 19–9 was related with malignant PCNs by univariate analysis and the other two researchers reported the opposite results.[24,27] The quality evaluation of the studies was shown in Fig 2. The risks of bias in “patient selection” were generally high in our meta-analysis, this represented the most prominent issue in the methodological quality assessment. Two studies with more than two high ‘risks’ were judged to be of low quality. The applicability was good.

**Table 1:** Characteristics of included studies.

Author,Year	Country	Study design	Mean age,years	Reference standards	No. of patients in studies	Patients included in meta-analysis	Patients with malignant disease	Patients with benign disease	Surgical pathology(n)	Cutoff vaule (u/ml)	Size of lesion
**Fritz et al., 2011**	**Germany**	**Retrospective**	**32–87**	**Pathology**	**142**	**142**	**50**	**92**	**142IPMNs: (Benign+HGD92, invasive50)**	**37**	**unknow**
**Goh et al., 2008**	**Canada**	**Retrospective**	**54(14–84)**	**Pathology**	**176**	**137**	**79**	**58**	**176PCNs: (Benign70, potentially malignant51, malignant55)**	**45**	**104patients>30mm,72patients<30mm**
**Grobmyer et al., 2009**	**USA**	**Retrospective**	**63**	**Pathology**	**78**	**40**	**8**	**32**	**78PCNs: (Non-malignant65, malignant13)**	**37**	**40mm**
**Hirono et al., 2012**	**Japan**	**Retrospective**	**68.9(32–84)**	**Pathology**	**134**	**134**	**78**	**56**	**134IPMNs: (Adenomas81, borderline neoplasms5, CIS41, invasive37)**	**elevated**	**30.4mm**
**Hwang et al., 2011**	**Korea**	**Retrospective**	**63(38–83)**	**Pathology**	**237**	**237**	**39**	**198**	**237IPMNs: (Aadenomas81, borderline117,CIS13,invasive26(**	**37**	**Malignant 36.6mm, unmalignant 25mm,**
**Ingkakul et al., 2010**	**Japan**	**Prospective**	**Malignant70, unmalignant66.4**	**Pathology,cyto-logy,follow-up**	**200**	**146**	**22**	**124**	200^*^IPMNs: (Invasive ductal carcinoma22, IPMNs alone93)	**37**	**unknow**
**Jones et al., 2009**	**USA**	**Retrospective**	**62(24–84)**	**Pathology**	**114**	**62**	**25**	**37**	**62PCNs: (Inasive35, IPMN29, MCN17, SMA33)**	**35**	**40mm**
**Kitagawa et al., 2003**	**USA**	**Retrospective**	**64**	**Pathology**	**63**	**42**	**21**	**21**	**63IPMN: (Benign30, invasive28, CIS5)**	**elevated**	**unknow**
**Ohtsuka et al., 2012**	**Japan**	**Retrospective**	**unknow**	**Pathology**	**138**	**99**	**22**	**77**	**99IPMNs: (LGD55, IGD22, HGD9, invasive13)**	**37**	**65patients>30mm, 34patients<30mm**
**Sadakari et al., 2010**	**Japan**	**Retrospective**	**66(46–82)**	**Pathology**	**73**	**53**	**6**	**47**	**73IPMNs: (Adenomas48, borderline19, CIS5, invasive1)**	**37**	**47patients>30mm, 26patients<30mm**
**Shin et al., 2010**	**Korea**	**Retrospective**	**61(35–77)**	**Pathology**	**204**	**195**	**49**	**146**	**204IPMNs: (Adenoma77, borderline77, CIS9, invasive41**	**37**	**85patients>30mm, 119patients<30mm**
**Sperti et al., 2007**	**Italy**	**Retrospective**	**64(37–84)**	**Pathology, follow-up**	**64**	**64**	**26**	**38**	64^**^IPMNs: (Adenomas13, borderline8, CIS5, invasive21)	**37**	**28mm**
**Xu et al., 2011**	**China**	**Retrospective**	**62(41–76)**	**Pathology**	**86**	**86**	**64**	**22**	**86IPMNs: (Adenomas10,borderline12,CIS3,invasive61)**	**37**	**unknow**

LGD: Low-grade dysplasia; IGD: Intermediate-grade dysplasia; HGD: High grade-dysplasia; CIS: carcinoma in situ. MCN: mucinous cystic neoplasms; SMA: serous microcystic adenoma.

* 85 patients did not undergo surgery

**17 patients did not undergo surgery

### Diagnostic performance

**Fig 3:**
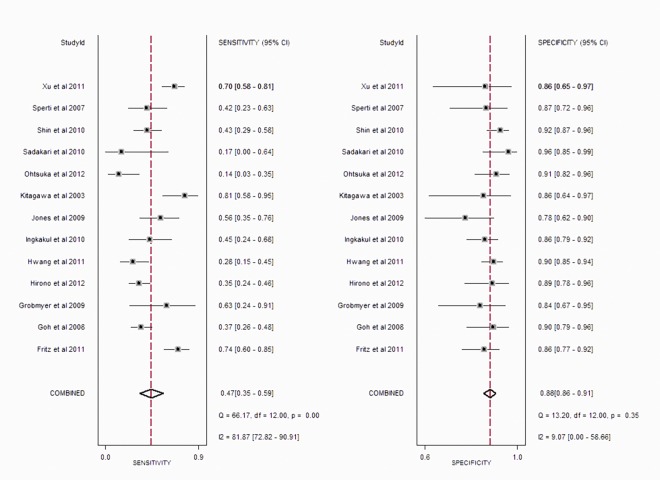
Forest plot of sensitivity and specificity estimates for serum CA 19–9 in predicting malignant PCNs for 13studies.

**Fig 4:**
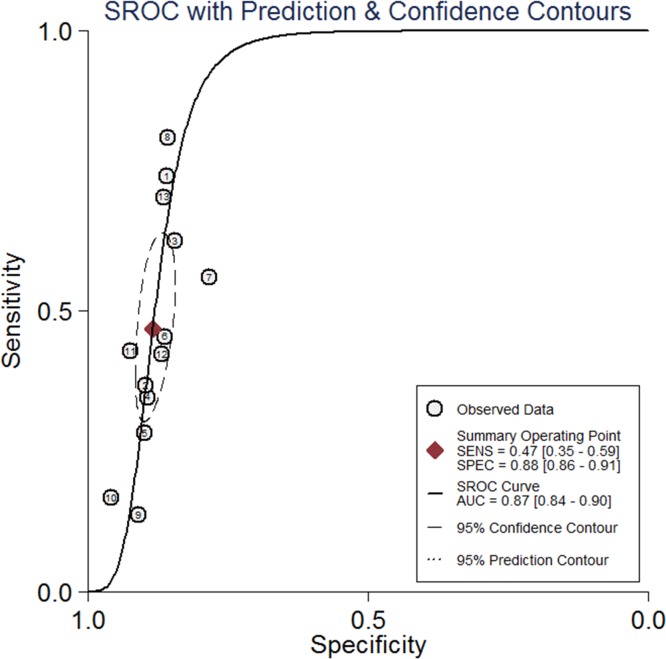
Summary of receiver operator characteristic (SROC) curve for serum CA 19–9 in predicting malignant PCNs. AUC: area under the curve.

The pooled sensitivity and specificity of serum CA 19–9 levels in predicting malignant PCNs were0.47 (95% CI: 0.35–0.59) and 0.88 (95% CI: 0.86–0.91), respectively (Fig 3). The positive likelihood ratio was 4.0 (95% CI, 3.2–5.2) and the negative likelihood ratio was 0.6 (95% CI, 0.48–0.75). The AUC was 0.87 (95% CI, 0.84–0.90) (Fig 4). The summary Diagnostic Odds Ratio (DOR) was 7 (95% CI: 4–12).

### Publications bias

**Fig 5:**
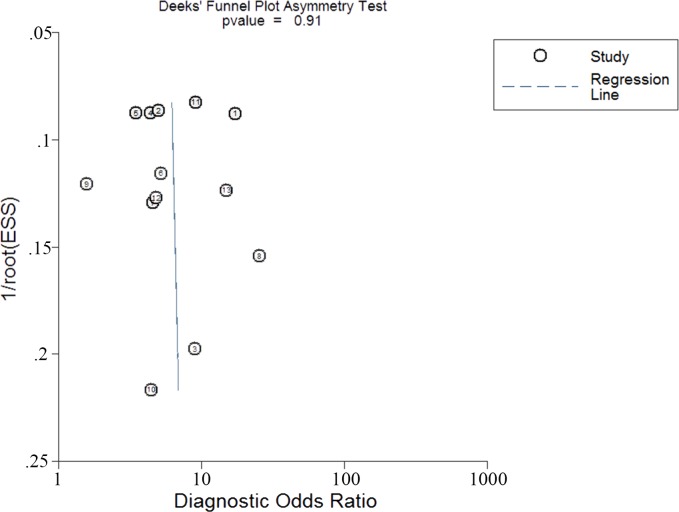
The Deek’s funnel asymmetry plot test for evaluation of potential publication bias for serum CA 19–9 in the diagnosis of malignant PCNs.

The publications bias of the studies was assessed by Deek funnel plot. The linear regression of log odds ratios on inverse root of effective sample sizes showed that the Bias value was 0.11 (*P* = 0.91), indicating no evidence of publications bias in these included studies (Fig 5).

### Heterogeneity assessing and subgroup analysis

Significant heterogeneity was observed in sensitivity (*P*<0.01; I^2^ = 81.87%), while no heterogeneity was showed in specificity (*P* = 0.35; I^2^ = 9.7%) among these 13 studies. Subgroup analysis was performed to identify possible reasons for the heterogeneity. We conducted subgroup analyses based on study sample size, study population and type of PCNs. There were six studies with sample size more than 100, involving 991 patients in total, which revealed a pooled sensitivity of 0.44 (95% CI: 0.32–0.56), specificity of 0.89 (95% CI: 0.86–0.91) and DOR of 6 (95% CI: 4–10), respectively. For studies with samples less than 100, the pooled sensitivity, specificity and DOR were 0.49 (95% CI: 0.28–0.69), 0.87 (95% CI: 0.81–0.91) and 6 (95% CI: 3–14), respectively. Seven studies were conducted in Asian patients, in which the pooled sensitivity, specificity and DOR were 0.37 (95% CI: 0.24–0.52), 0.90 (95% CI: 0.87–0.92) and 5 (95% CI: 3–10), respectively. The remaining 6 studies, conducted in patients from the western countries, showed a pooled sensitivity of 0.59 (95% CI: 0.44–0.72), specificity of 0.86 (95% CI: 0.81–0.89) and DOR of 8 (95% CI: 4–16), respectively. There were 10 studies involving patients with IPMNs only, whose pooled sensitivity and specificity were 0.45 (95% CI: 0.31–0.61), 0.89 (95% CI: 0.86–0.91), respectively. The remaining 3 studies were insufficient for analysis.

**Fig 6:**
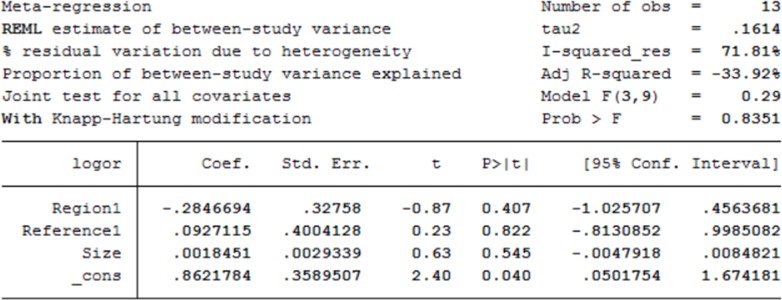
The meta-regression analysis for possible resources of heterogeneity among included studies.

For meta-regression analysis, we found that these pre-identified confounding covariates, like sample size, region, reference standards may cause heterogeneity. But these factors were not the main sources of heterogeneity as shown in Fig 6.

## Discussion

There are growing number of cystic lesions identified in pancreas due to the increasingly wide use of abdominal imaging.[28] Correct preoperative diagnosis is the pillar of clinical management.[5,29] It has been reported that preoperative factors, such as serum tumor markers, cyst fluid tumor markers, tumor size, diameter of the main pancreatic duct, symptoms, duct type, EUS-FNA-based cytology and cross-sectional imaging features are reasonably accurate in predicting the malignant potential of PCNs.[15,25,30–35] Several articles reported that elevated serum CA 19–9 is a significant high risk factor of malignant PCNs. [15,16,20,25,26]

In our meta-analyses, we summarized 13 studies involving 1437 patients who were diagnosed with PCNs. To predict malignancy of PCNS, the pooled specificity and AUC of serum CA 19–9 were satisfactory, while the pooled sensitivity was limited. This might suggest that serum CA 19–9 would be a useful discriminator between benign and malignant PCNs, but more sensitive biomarkers were needed. A significant number of malignant PCNs might be misdiagnosed as benign disease when only relying on serum CA 19–9. Our viewpoint was also agreed by many other researchers, who reported that serum CA 19–9 was a useful non-invasive tool for distinguishing malignant IPMNs, and should be taken into account in the decision to offer surgery.[15,17,36]

Many other diagnostic techniques were also used to distinguish benign and malignant PCNs. It was illuminated that the risk of malignant PCNs associated with cyst size greater than 3cm, presence of a mural nodule, dilatation of the main pancreatic duct, etc.[37] A meta-analysis reported that the level of cyst fluid CEA would not differentiate benign from malignant PCNs well, with a pooled sensitivity (0.63; 95% CI: 0.55–0.69) and specificity (0.63; 95% CI: 0.57–0.68), respectively.[38] The international consensus guidelines 2012 for PCNs identified that elevated CEA of cyst fluid was not an effective biomarker to distinguish benign from malignant cysts. [5] Suzuki analyzed EUS-FNA-based cytology differentiating malignant and benign IPMNs. Similar to our diagnostic test, the pooled sensitivity and specificity were 0.64 (95% CI, 0.44–0.82) and 0.9 (95% CI, 0.81–0.96), respectively.[39] But it is more safe, convenient and economic to detect serum CA 19–9 in clinical practice. Sultana reported that the pooled sensitivity and specificity of CT/MRI in distinguishing malignant IPMNs were 0.89 (95% CI, 0.71–0.88), 0.76 (95% CI, 0.65–0.85), respectively.18-fluorodeoxyglucose positron emission tomography (18-FDG PET) is believed to be better than other imaging technology in distinguishing benign from malignant IPMNs.[23,40] The pooled sensitivity and specificity of 18-FDG PET were 0.968 (95% CI: 0.90–0.99) and 0.91(0.81–0.99).[36] As 18-FDG PET is not routinely performed for its high cost and radiation, the decision of whether to do a surgery or not remains challenging.[41] Studies of molecular markers were also reported recently. It is reported that the sensitivity and specificity of KRAS mutations analysis in differentiating malignancy from benign PCNs were 0.59 (95% CI: 0.46–0.71) and 0.78(0.71–0.85), respectively. And the pooled sensitivity and specificity of loss of heterozygosity of KRAS gene were 0.89 (95% CI: 0.78–0.96), 0.69 (95% CI: 0.60–0.76), respectively.[42] Compared with these tests, serum CA19-9 can be an initiatory and essential preoperative assessment in routine clinical practice. According to the pooled specificity and AUC in our meta-analysis, it’s helpful to preliminarily identify malignant PCNs. As it is impossible to establish a diagnosis of malignant PCNs before surgery with one unique inspection technique, a combined preoperative diagnosis strategy with multi-disciplinary approach may be more promising.

There are several limitations in our meta-analysis. First, high heterogeneity was found in our meta-analysis and the meta-regression analyses failed to explore the main resources of heterogeneity among studies. The possible reason is that studies shared different cutoff values or definitions of malignant PCNs, which might not be taken into account in our statistical model. The various criteria used to evaluate malignant PCNs, such as CIS, HGD, potentially malignant cancer, invasive cancer and malignant cancer among studies, may result in heterogeneity. In addition, the term CIS has been suggested to be abandoned in the international consensus guidelines. [5] Second, most of the included studies didn’t provide the information of assays used to detect serum CA 19–9, and such differences in detection may be an important source of heterogeneity. Third, only studies published in English were included in our meta-analysis, which may also result in bias. Finally, we just valued the diagnostic precision of serum CA 19–9 in discriminating benign from malignant PCNs, but failed to deeply discuss what should we manage patients with normal or evaluated CA 19–9.

## Conclusions

In conclusion, this meta-analysis indicated that serum CA 19–9 could be a useful tool with high pooled specificity for discriminating benign from malignant PCNs. Considered as the most economic, convenient, safe and rapid method,it deserved to be widely used as complementary to other diagnostic techniques. There is still a need for further studies looking into features associated with malignant PCNs.

## Supporting Information

S1 PRISMA Checklist Prisma Harms checklist items. (DOCX)
